# Sensing Exocytosis and Triggering Endocytosis at Synapses: Synaptic Vesicle Exocytosis–Endocytosis Coupling

**DOI:** 10.3389/fncel.2018.00066

**Published:** 2018-03-14

**Authors:** Xuelin Lou

**Affiliations:** Department of Cell Biology, Neurobiology and Anatomy, Medical College of Wisconsin, Milwaukee, WI, United States

**Keywords:** exocytosis-endocytosis coupling, active zone, endocytic zone, sensors, dynamin, membrane tension

## Abstract

The intact synaptic structure is critical for information processing in neural circuits. During synaptic transmission, rapid vesicle exocytosis increases the size of never terminals and endocytosis counteracts the increase. Accumulating evidence suggests that SV exocytosis and endocytosis are tightly connected in time and space during SV recycling, and this process is essential for synaptic function and structural stability. Research in the past has illustrated the molecular details of synaptic vesicle (SV) exocytosis and endocytosis; however, the mechanisms that timely connect these two fundamental events are poorly understood at central synapses. Here we discuss recent progress in SV recycling and summarize several emerging mechanisms by which synapses can “sense” the occurrence of exocytosis and timely initiate compensatory endocytosis. They include Ca^2+^ sensing, SV proteins sensing, and local membrane stress sensing. In addition, the spatial organization of endocytic zones adjacent to active zones provides a structural basis for efficient coupling between SV exocytosis and endocytosis. Through linking different endocytosis pathways with SV fusion, these mechanisms ensure necessary plasticity and robustness of nerve terminals to meet diverse physiological needs.

## Introduction

Synaptic transmission is fundamental to brain function. Nerve terminals release neurotransmitter at a different speed, depending on the types of synapses and stimulation. The rate of synaptic vesicle (SV) exocytosis directly controls neurotransmission strength. It can vary a few orders of magnitude at a single terminal (from <1 Hz up to ~1,000 Hz) (Lou et al., 2005) and thus provide a large dynamic range of synaptic transmission (Schneggenburger and Rosenmund, 2015). Depending on release probability and readily releasable vesicle pool (RRP) size, a presynaptic terminal can rapidly release numerous SVs during a brief train of action potentials (APs) (Neher, 2015).

**Figure 1:**
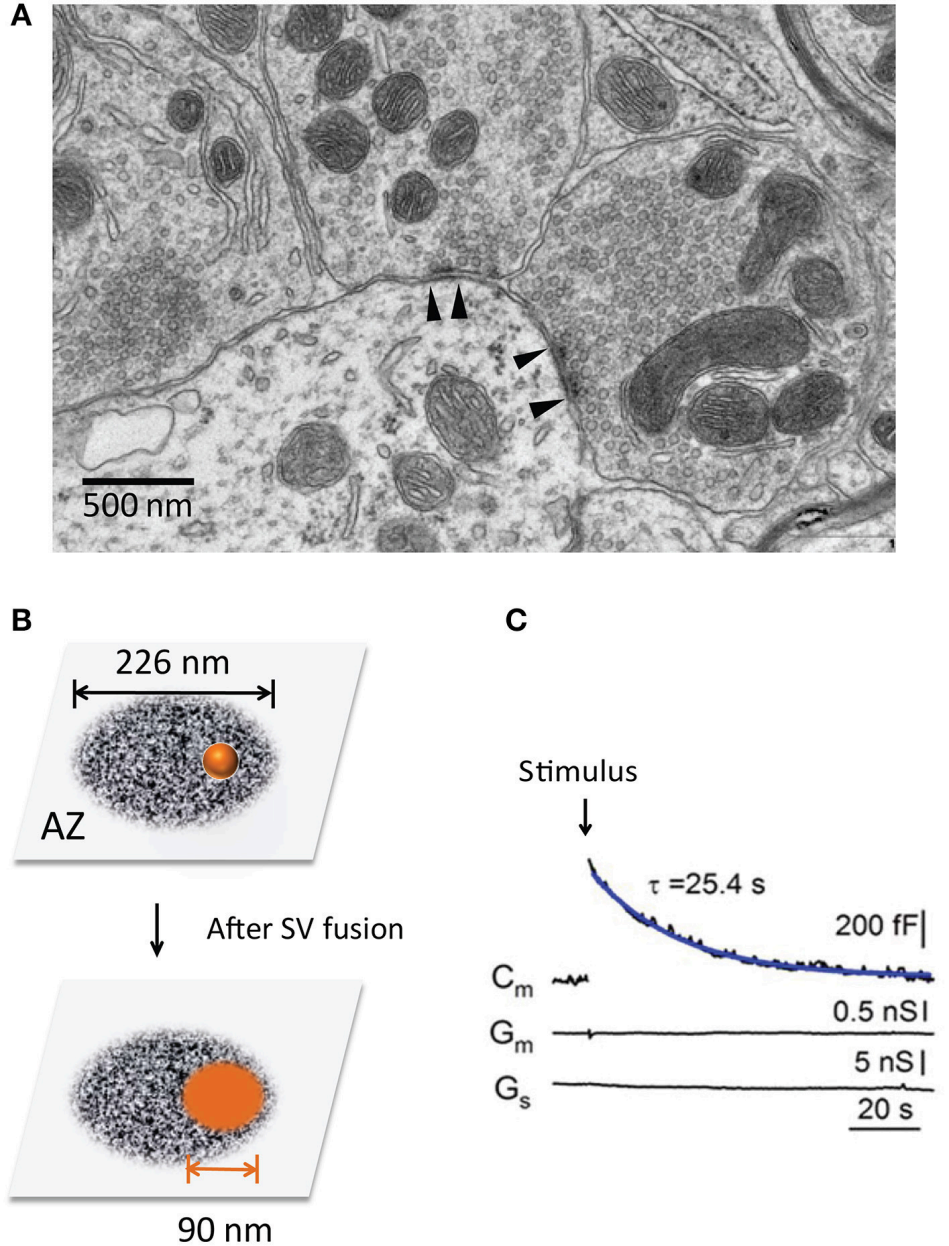
The transient expansion of the presynaptic surface area. **(A)** Ultrastructure of the presynaptic nerve terminals in a mouse cerebellum (chemical fixation). Note two AZs with high electronic density (between the arrowheads). **(B)** The surface area of a single SV after it fuses and merges with the PM at an AZ. The scheme size is shown in scale, the AZ area is 0.4 μm^2^ (Holderith et al., 2012) and SV diameter is 45 nm (Lou et al., 2008). **(C)** Transient changes of the surface area are recorded at the calyx of Held during a 20 ms depolarization pulse (arrow) (Lou et al., 2008). Note the rapid increase and subsequent recovery of Cm, suggesting SV fusion and compensatory endocytosis.

Given the smaller size of AZs, each SV fusion can significantly expand the plasma membrane (PM) of AZs and thus impact its ultrastructure and function (Figure 1). Remarkably, synapses are capable to counteract this structural change by endocytosis in a timely fashion (Ceccarelli et al., 1972; Heuser and Reese, 1973). The temporal coupling between exocytosis and endocytosis ensure the structural stability and functional integrity of chemical synapses during active SV recycling.

Decades of research have generated a wealth of knowledge on the molecular mechanisms of SV exocytosis (Jahn and Fasshauer, 2012; Südhof, 2013; Herman and Rosenmund, 2015) and endocytosis (Saheki and De Camilli, 2012; Wu L. G. et al., 2014; Soykan et al., 2016). However, exactly how synapses timely coordinate these two fundamental events remains largely ambiguous. SV exocytosis, which occurs within a millisecond, is much faster than any type of SV endocytosis reported so far. During rapid neurotransmission, repeated SV release places an important task for synapses to avoid SV traffic jam. The presence of synaptic active zones (AZs), a highly-specialized structure that regulates SV trafficking (recruitment, docking, fusion and coupling to endocytosis), makes the exo-endocytosis coupling of SVs more complex than dense core vesicles (DCVs) in other types secretory cells (Liang et al., 2017; Neher, 2018). This paper focuses primarily on SV exocytosis-endocytosis coupling at the presynaptic nerve terminals.

## SV exocytosis–endocytosis coupling at nerve terminals

### Prevalence

SV exocytosis–endocytosis coupling exists at a variety of nerve terminals. The direct evidence in living synapses comes from two types of experiments. One is capacitance recordings, which monitor cell surface area (Neher and Marty, 1982). The presynaptic membrane capacitance (Cm) shows a depolarization-triggered Cm increase and a subsequent recovery to baseline (Sun and Wu, 2001; Lou et al., 2008), indicating SV fusion with the PM and an equal amount of membrane retrieval afterward (Figure 1C). The PM expansion is transient in response to a short pulse but lasts longer during continuous stimulation, indicating the net balance of exocytosis and endocytosis. Similar Cm changes have been reported at other types of nerve terminals, such as ribbon synapses in the retina (von Gersdorff and Matthews, 1994; Neves and Lagnado, 1999) and hair cells (Moser and Beutner, 2000), mossy fiber boutons in the hippocampus (Hallermann et al., 2003) and cerebellum (Delvendahl et al., 2016). The second evidence is from interference reflection microscopy at living ribbon synapses. Direct imaging demonstrates rapid cell surface expansion and subsequent recovery that matches with the Cm responses (Llobet et al., 2003).

Optical imaging with pH-sensors also supports the tight exocytosis-endocytosis coupling. At individual synapse levels, APs trigger a rise-and-fall of fluorescence from pHluorin-tagged SV proteins (Sankaranarayanan and Ryan, 2000). At the single SV level, the timely coupling is also demonstrated by VAMP2-pHluorin (Gandhi and Stevens, 2003), quantal dots (Zhang et al., 2009), synaptophysin-pHluorin (Granseth et al., 2006; Zhu et al., 2009), and vGlut-pHluorin (Balaji and Ryan, 2007; Leitz and Kavalali, 2014). Similarly, cypHer-tagged probes produce a mirror response (fall-and-rise) to pHluorin sensors because of its opposite pH sensitivity (Hua et al., 2013). These data suggest a tight balance between SV exocytosis and endocytosis at central synaptic terminals.

In addition, electron microscopy (EM) studies support the ultrastructure changes of motor nerve terminals in frog neuromuscular junctions (NMJs) (Ceccarelli et al., 1972; Heuser and Reese, 1973). The quick-freezing EM examines the nerve terminals at different intervals post an AP and demonstrates the brief surface expansion and subsequent recovery (Miller and Heuser, 1984), with an AZ ultrastructure recovery within ~90 s. A recent flash-freezing EM study in hippocampus cultures reported a much fast coupling (within ~100 ms) between SV exocytosis and endocytosis. On average, ~0.7 SV exocytosis is coupled with ~0.6 SV-equivalent membrane retrieval per synapse (Watanabe et al., 2013b). Cortical synapses are able to maintain their size both in cultures (Hayashi et al., 2008) and intact brain circuitry (Lou et al., 2008) even in the absence of dynamin-1, a protein involved in vesicle fission. Under strong stimulation, bulk endocytosis is upregulated about 2-fold in dynamin KO synapses to counteract the nerve terminal expansion (Hayashi et al., 2008; Wu Y. et al., 2014).

### Function

The timely coupling of exocytosis and endocytosis has a profound impact on synaptic structure and function. First, it preserves the overall size of nerve terminals. Second, it recycles SV components that are required for new SV production (Dittman and Ryan, 2009). Long-term neurotransmission relies on endocytosis and SV recycling, even though RRP and reserve pool SVs can support short-term neural activity (Rizzoli and Betz, 2005; Neher, 2015). In addition, only a very small portion of total SVs participate actively in vesicle recycling during sustained transmission (Denker et al., 2011), adding another layer of urgency for rapid SV regeneration.

Third, the timely retrieval of SV components is important for release-site clearance (Neher, 2010). The limited release-sites must be re-used during sustained neurotransmission. Each SV fusion leads to two basic impacts: (1) vesicle components (e.g., SV proteins Takamori et al., 2006, used SNAREs) occupy release sites and need to be cleared away rapidly (Neher, 2010). The efficient clearance would make release-site re-competent for a new round of vesicle fusion, and this process may become rate-limiting during fast neurotransmission (Neher, 2010; Hua et al., 2013; Mahapatra et al., 2016). 2) ultrastructure change of AZs. Given the average size of SVs (~45 nm in diameter) (Lou et al., 2008) and AZs (~0.04 μm^2^, ranging from 0.02 ~ 0.2 μm^2^) (Han et al., 2011; Holderith et al., 2012) (Figure 1), each SV fusion adds an additional membrane area of ~6,362 nm^2^ (*A* = 4 π *r*^2^), ~16% of the original AZ area (Figure 1B). This expansion can alter AZ nanostructure and release-site organization (t-SNARE, complexin, Munc-13, Ca^2+^ channels, etc). The quick-freezing EM study in NMJs (Heuser and Reese, 1981) has captured such effects. These two impacts may become prominent during repeated SV fusion since most SVs fuse at AZs (Figure 1A) rather than randomly. Thus, tight SV exocytosis-endocytosis coupling helps to maintain AZ ultrastructure integrity.

## Multiple modes of SV exocytosis-endocytosis coupling

Four types of endocytosis are proposed at synapses: “kiss and run” (K&R), clathrin-mediated endocytosis (CME), bulk endocytosis, and ultrafast endocytosis (for details, see Saheki and De Camilli, 2012; Alabi and Tsien, 2013; Wu L. G. et al., 2014; Soykan et al., 2016; Watanabe and Boucrot, 2017). None of them alone can account for all the experimental observations at synapses in literature. Each endocytosis route likely couples differently with exocytosis events, depending on neural activity as well as synapse types.

First, endocytosis couples with exocytosis at the same site via K&R (or Kiss-and-stay). An SV fuses with the PM and pinches off at the same location without collapsing (Alabi and Tsien, 2013). This mechanism is first proposed by Ceccarelli and colleague based on EM studies (Ceccarelli et al., 1972), and it remains highly debatable since then. K&R at synapses completes within ~0.5 s (Gandhi and Stevens, 2003; Zhang et al., 2009) or ~0.3 s (He et al., 2006). This coupling mode is thought to promote rapid SV recycling because of its fast speed than CME.

Second, SVs fuse at AZs and are retrieved by CME at endocytic zones (EZs) (Dittman and Ryan, 2009; Saheki and De Camilli, 2012). CME is proposed by Heuser and Reese (Heuser and Reese, 1973, 1981) and subsequently investigated extensively (Dittman and Ryan, 2009; Saheki and De Camilli, 2012). It operates at a time constant (τ) of ~15 −20 s according to pHluorin assays (Granseth et al., 2006; Balaji and Ryan, 2007), consistent with the Cm assays at the calyx of Held (τ = ~10–25 s) (Wu et al., 2005; Yamashita et al., 2005; Lou et al., 2008). CME accelerates at physiological temperature (PT, 37°C) (Renden and von Gersdorff, 2007; Wu et al., 2016) as compared to room temperature (RT), despite the estimated endocytosis rate varies largely among different research groups [e.g., τ = ~3 s (Leitz and Kavalali, 2011), 6 s (Balaji and Ryan, 2007; Armbruster et al., 2013), and 20 s (Soykan et al., 2017)]. Multiple lines of evidence support that CME is a dominant endocytosis pathway at nerve terminals (Heuser and Reese, 1973; Granseth et al., 2006; Dittman and Ryan, 2009; Saheki and De Camilli, 2012). For example, typical SV endocytosis is severely impaired after clathrin-knockdown (Granseth et al., 2006; Nicholson-Fish et al., 2015) and AP-2 α-μ2 double mutants in *C. elegans* (Gu et al., 2013). CME is also critical in squid giant synapses (10–15°C) (Augustine et al., 2006), whose physiological temperature is low; perturbations of clathrin assembly (or uncoating) showed a loss of SVs and expansion of presynaptic PM area (Morgan et al., 1999, 2000). However, this notion is challenged by recent studies (Sato et al., 2009; Kononenko et al., 2014; Watanabe et al., 2014; Delvendahl et al., 2016; Soykan et al., 2017). This is a critical question especially for mammals in which synapses operate routinely at 37°C. Therefore, more work is required to address SV CME under physiological condition.

Third, SV fusion couples with bulk endocytosis. Bulk endocytosis is observed frequently under EM, where it displays as large membrane vacuoles with variable sizes (~80–300 nm in diameter) (Miller and Heuser, 1984; Hayashi et al., 2008; Wu Y. et al., 2014). Further EM tomography demonstrates that those vacuole structures are unconnected with the presynaptic PM (Hayashi et al., 2008). Bulk endocytosis requires VAMP-4 (Nicholson-Fish et al., 2015) and F-actin (Shupliakov et al., 2002; Holt et al., 2003), and it occurs mainly during high-frequency APs as shown in neuronal cultures (Clayton et al., 2008; Wu Y. et al., 2014). Bulk endocytosis has a higher capacity to retrieve the PM (Shupliakov et al., 2002; Wu and Wu, 2007; Lou et al., 2008) but possesses a poorer cargo-selectivity and quality control than CME (Miller and Heuser, 1984; Nicholson-Fish et al., 2015). In addition, some large PM cisterns are also observed 15 min after stimulation in frog NMJs (Heuser and Reese, 1973), indicating a delayed or different form of bulk endocytosis. It is unclear whether this type of bulk endocytosis is relevant to the step-like Cm decrease recorded at the calyx of Held (Wu and Wu, 2007; Lou et al., 2008). Bulk endocytosis likely serves as an emergency endocytosis route for synapses to counteract their surface expansion during high neural activity.

Finally, SV fusion couples with ultrafast endocytosis. Ultrafast endocytosis is reported by Watanabe et al using high-pressure freezing EM in combination with optogenetic stimulation (Watanabe et al., 2013a,b). Synapses expressing channelrhodopsin are stimulated with a blue light to trigger neurotransmitter release. Ultrafast endocytosis peaks at 50–100 ms of stimulation and generates uncoated vesicles with a uniform size (~82 nm in diameter, ~2-fold larger than SVs) (Watanabe et al., 2013b, 2014). Ultrafast endocytosis operates selectively at 34 ~ 37°C (but fails at 20°C) in mammalian synapses (Watanabe et al., 2014) although it also appears at RT in motor nerve terminals of *C. elegans* (Watanabe et al., 2013a) (whose cultivation temperature spans 15 ~ 25°C). Ultrafast endocytosis requires SV fusion and F-actin. It is also sensitive to dynasore, a dynamin inhibitor, but its effect on F-actin and other targets (Park et al., 2013; Mahapatra et al., 2016) should be carefully considered. This new form of SV exocytosis-endocytosis coupling adds a new layer of complexity to SV recycling.

Interestingly, in the time-resolved quick-freezing EM study (with a single AP stimulation at RT) (Heuser and Reese, 1981), Heuser and Reese have reported both CME and “a second form of membrane retrieval.” The latter operates through “a random bite” of a large piece of plasma membrane without clathrin-coat, similar to bulk endocytosis. Moreover, it occurs “in the first a few milliseconds after stimulation” (Heuser and Reese, 1981), similar to (if not faster than) the ultrafast endocytosis under flash-freezing EM at PT. It is unclear whether they are the same form of endocytosis.

While the details of CME have been addressed, the mechanism for other forms of endocytosis remains poorly understood. Future work on molecular characterization may help to better define these different forms of endocytosis. Ultrafast endocytosis and bulk endocytosis exhibit different properties (in speed, retrieval size, temperature sensitivity, and stimulation strength to trigger them), but they also have some common features (e.g., high capacity, clathrin-independence, F-actin dependence, and endosome sorting).

## Sensors and triggers for endocytosis in the coupling

Despite the significant progress on SV exocytosis as well as endocytosis, very little is known about their temporal and spatial coupling. What triggers SV endocytosis at the right time and place remains a longstanding question. Recent work suggests that several factors are involved in SV exocytosis-endocytosis coupling, in which a synapse “senses” the SV exocytosis event and initiates endocytosis. Here, we discuss the potential means of coupling at nerve terminals.

### Ca^2+^ and Ca^2+^ sensors

**Figure 2:**
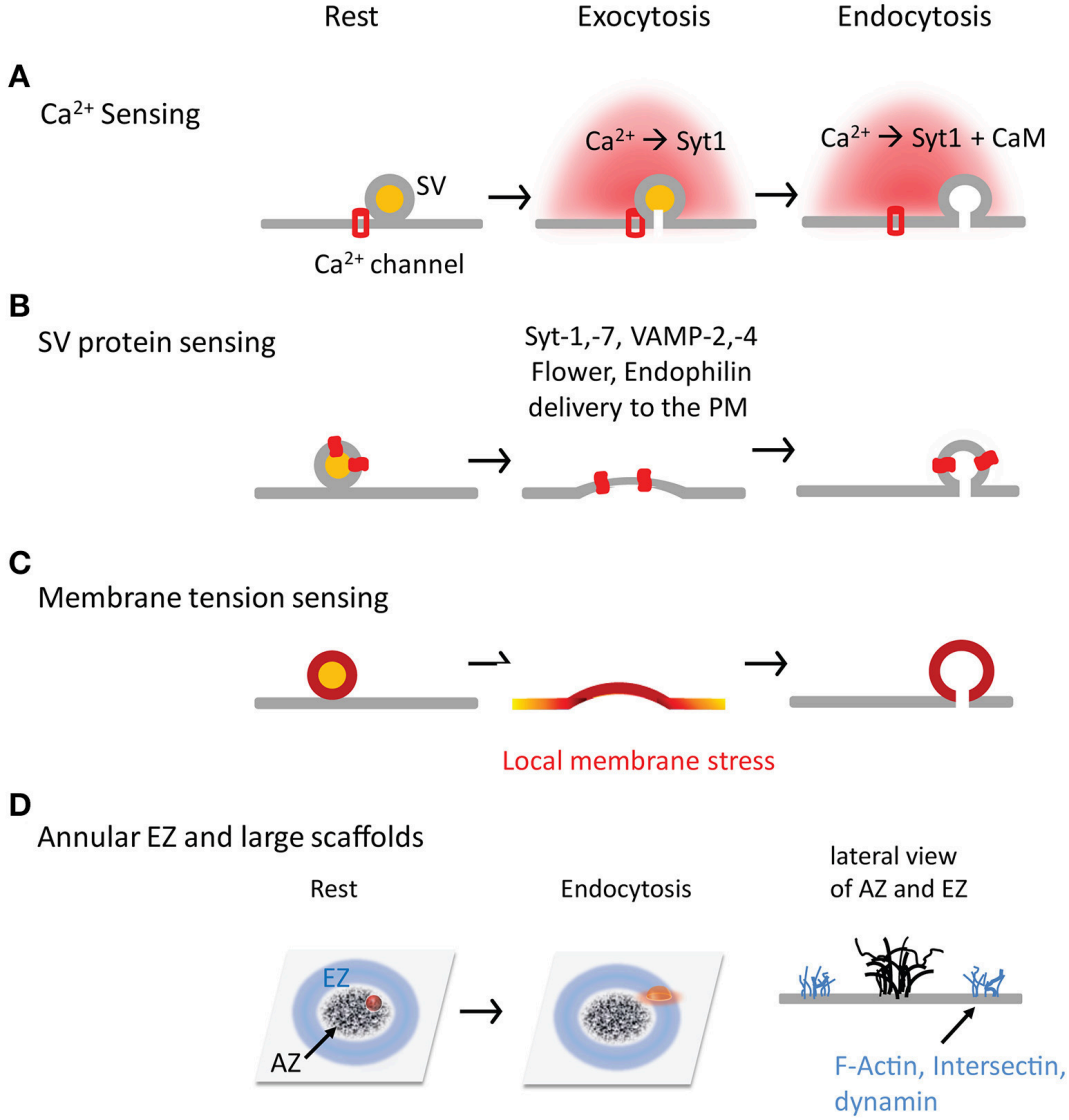
Four hypothetical mechanisms by which synapses sense exocytosis and initiate endocytosis in time and space. **(A)** Ca^2+^ is a trigger, and synaptotagmin and CaM are sensors in the SV exocytosis-endocytosis coupling. **(B)** SV fusion is a trigger, and SV proteins (both transmembrane and associated proteins) are sensors to initiate CME by interacting with AP-2. **(C)** SV fusion is a trigger to generate the local membrane stress. **(D)** The annular spatial arrangement of EZs surrounding AZs. The large scaffolds are critical for SV exocytosis-endocytosis coupling by facilitating SV proteins transportation and recapture.

Intracellular Ca^2+^ classically regulates both exocytosis and endocytosis (Neher and Sakaba, 2008) and is therefore a suitable candidate for the coupling (Figure 2A). Intracellular Ca^2+^ micro- or nano-domains (Neher, 1998) are generated during an AP due to the uneven distribution of Ca^2+^ channels at AZs (Althof et al., 2015; Nakamura et al., 2015). While the local Ca^2+^ domains tightly regulate SV fusion (Eggermann et al., 2011; Schneggenburger et al., 2012), they are also required in SV endocytosis as demonstrated at the mature calyx of Held (Yamashita et al., 2010). Ca^2+^ uncaging experiments suggest that a ~15 μM Ca^2+^ increase is needed to trigger rapid endocytosis in inner hair cells, despite endocytosis remaining constant (τ = 16 s) below that Ca^2+^ level (Beutner et al., 2001). A minimal of ~10 μM Ca^2+^ is necessary to initiate endocytosis at the calyx of Held (Hosoi et al., 2009), consistent with the result (~11 μM) from lamprey reticulospinal synapses (Gad et al., 1998). These Ca^2+^ values are as high as that for triggering SV fusion (Bollmann et al., 2000; Schneggenburger and Neher, 2000; Lou et al., 2005; Neher and Sakaba, 2008; Kochubey et al., 2011), implying that endocytosis occurs much closer to Ca^2+^ channels than previously thought. Accordingly, AP-2 is shown to interact with Ca^2+^ channels at the synprint region (Watanabe et al., 2010). Therefore, intracellular Ca^2+^ is likely a trigger for endocytosis.

Following Ca^2+^ elevation, synaptotagmin acts as a Ca^2+^ sensor for endocytosis, similar to its function in exocytosis (Geppert et al., 1994; Chapman, 2008). Genetic perturbations of synaptotagmin severely impair SV endocytosis (Poskanzer et al., 2003; Llinás et al., 2004; Nicholson-Tomishima and Ryan, 2004; Yao et al., 2012). The Ca^2+^ sensing property of synaptotagmin is critical for this effect (Poskanzer et al., 2006; Yao et al., 2011). It is reported that different Ca^2+^ binding affinity of Ca^2+^ sensors (e.g., synaptotagmin-1 and−7) allows differential endocytic regulation under weak and strong stimulations (Li et al., 2017). Similarly, Ca^2+^ sensor otoferlin is found to regulate endocytosis in hair cells (Duncker et al., 2013).

In addition to synaptotagmin and otoferlin, calmodulin (CaM) is another Ca^2+^ sensor for endocytosis (Figure 2A). Intracellular Ca^2+^ elevation activates CaM-calcineurin to dephosphorylate dephosphins, a group of endocytosis proteins that are constitutively phosphorylated at rest (Cousin and Robinson, 2001). Interruptions of CaM (Artalejo et al., 1996; Wu et al., 2009; Yao and Sakaba, 2012) and calcineurin (Marks and McMahon, 1998; Sun et al., 2010; Yamashita et al., 2010; Wu X. S. et al., 2014) inhibit endocytosis, although results vary with stimulation conditions (Yao and Sakaba, 2012) and age (Yamashita et al., 2010). Dynamin-1 (Serines at 774 and Ser 778) dephosphorylation and phosphorylation mutations abolish the biphasic regulation of Ca^2+^ on SV endocytosis (Armbruster et al., 2013). This effect depends on dynamin interactions with syndapin-1 rather than endophilin or synaptophysin (Anggono et al., 2006). After the neural activity, intracellular Ca^2+^ recovery and CaM-calcineurin activity decreases; meanwhile cyclin-dependent kinase 5 (Cdk5) rephosphorylates dephosphins to terminate endocytosis (Tan et al., 2003). This CaM-calcineurin/Cdk5 balance offers another way of Ca^2+^-sensing in SV exocytosis-endocytosis coupling. In addition, it is reported that CaM interacts with N-BAR proteins Rvs167 (in yeast), amphiphysin and endophilin-A (Myers et al., 2016), suggesting a calcineurin-independent regulation of CaM on endocytosis.

Two relevant questions are noteworthy. First, Ca^2+^ elevation alone appears insufficient in triggering endocytosis, as demonstrated by the experiment in munc13-1 and−2 double KO synapses (Watanabe et al., 2013b). Accordingly, perturbations of Ca^2+^ downstream molecules (e.g., synaptotagmin, CaM, and calcineurin) do not abolish endocytosis in many studies. High sucrose stimulation also triggers efficient endocytosis (Yao et al., 2011). These data suggest that Ca^2+^ needs to work with other factors. Second, Ca^2+^ has a more complex role in endocytosis than in exocytosis (Leitz and Kavalali, 2016). For example, local Ca^2+^ domains accelerate endocytosis but a global dialysis of Ca^2+^ inhibits it (Wu and Wu, 2014); similar counteracting effects also occurs at the same synapses under different conditions (Armbruster et al., 2013): accelerating endocytosis under moderate stimulation and slowing it down under strong stimulation.

### SV proteins

There are a large number of proteins on individual SVs. After SV fusion, some of SV proteins that newly added to the PM are suitable candidates for SV exocytosis-endocytosis coupling, which allow synapses to “sense” SV fusion events and initiate endocytosis in a timely fashion (Figure 2B).

#### Synaptotagmin-1

This molecule has a high copy number on each SV (15 copies/SV) (Takamori et al., 2006). After its delivery to the PM, synaptotagmin serves as a nucleating factor to recruit AP-2 (Haucke and De Camilli, 1999), a key component of CME. it can directly interact with clathrin-associated sorting protein stonin-2 (Jung et al., 2007), adaptor protein AP-2 (Willox and Royle, 2012) and SV2A/B (Kaempf et al., 2015). Consequently, synaptotagmin shows dual roles in SV endocytosis: sensing Ca^2+^ and nucleating CME components.

#### SNARE proteins

VAMP2 (also called Synaptobrevin-2) is a core protein of SNARE complex and has the highest copy number (~70 copies/SV) on SVs (Takamori et al., 2006). After VAMP-2 interruptions, endocytosis is impaired as shown in experiments using VAMP-2 KO (Deák et al., 2004), knockdown (Zhang et al., 2013) and its blocking peptide or cleavage toxin (tetanus toxin) (Hosoi et al., 2009; Xu et al., 2013). VAMP-4 is reported to regulate bulk endocytosis selectively (Nicholson-Fish et al., 2015). Interestingly, other components of SNARE machinery have also been reported to regulate endocytosis, including syntaxin1a (Xu et al., 2013), SNAP-25 (Xu et al., 2013; Zhang et al., 2013), and complexin (Li et al., 2017). Syntaxin1a and SNAP-25 probably play a permissive role rather than acting as a trigger in SV endocytosis, since they are already present abundantly on the PM before SV fusion.

#### Synaptophysin-1, endophilin, and vGlut-1

Synaptophysin-1 has the second highest copy number on SVs (32 copies/SV) and resides exclusively on SVs. Both properties make it a suited endocytosis sensor. Synaptophysin-1 KO blocks VAMP-2 endocytosis (Gordon et al., 2011) in a stimulation- and frequency-dependent manner (e.g., 200 AP at 20 Hz) (Kwon and Chapman, 2011; Rajappa et al., 2016). Endophilin plays a role in CME and SV uncoating (Milosevic et al., 2011); it's activity-dependent sub-synaptic translocation (from SV clusters to EZs) in C-elegant DA neurons (Bai et al., 2010) indicates its potential role in facilitating efficient coupling. Moreover, endophilin can bind dynamin, synaptojanin (Milosevic et al., 2011), and vGlut-1 (Voglmaier et al., 2006), and thus accelerates CME. Knockdown of vGlut-1 also slows down endocytosis of SV2 and synaptophysin, suggesting a new role of vGlut-1 in regulating cargo sorting and endocytosis during CME (Pan et al., 2015).

#### Flower

This is an SV-associated protein with Ca^2+^-permeable channel property (Yao et al., 2009). Once inserting in the PM after SV fusion, Flower is an ideal factor to generate local Ca^2+^ elevation for endocytosis. The flower was identified by a forward genetic screening in *Drosophila*, in which Flower mutants exhibited impaired endocytosis and basal Ca^2+^ (Yao et al., 2009). It is able to form Ca^2+^ permeable channels *in vitro* and to increase intracellular Ca^2+^ concentration when expressed in salivary gland cells. This phenomenon is similar to the case of P-type Ca^2+^ channels in sea urchin eggs (Smith et al., 2000), in which the channels on secretory granules insert in the PM after exocytosis and determine the endocytosis location. However, the channel activity of Flower is undetectable at the calyx of Held synapses (Xue et al., 2012); its Ca^2+^ permeability appears to be critical selectively for bulk endocytosis (Yao et al., 2017).

### The large scaffolds that bridge AZs and EZs

SV fusion and endocytosis occur at adjacent PM domains: AZs and EZs, respectively. The AZ is a tiny presynaptic area with high electron-density (Gundelfinger and Fejtova, 2012). AZs contain a set of large, multiple-domain proteins (called the cytomatrix of AZs, CAZs), including CAST/ELKS/Bruchpilot protein (Brp), Liprin-α, Rab3-interacting molecules (RIMs), RIM-binding proteins (RIM-BPs), Bassoon, Piccolo/Aczonin, and UNC-13/Munc-13 (Gundelfinger and Fejtova, 2012; Südhof, 2012; Ackermann et al., 2015). CAZs control SV dynamics and release probability by regulating AZ ultrastructure. Genetic perturbations of key genes encoding CAZ proteins impair neurotransmission (Südhof, 2012; Ackermann et al., 2015). For example, Brp mutation in fly NMJ causes AZ (T-bar) disassembly, Ca^2+^ channel cluster loss, and exocytosis defect (Fouquet et al., 2009). ELSK and RIM double KO synapses in mice display similar phenotypes: AZ disassembly, lack of docked SVs, decreased transmitter release, and degradation of other CAZ proteins (Wang et al., 2016). In addition to functioning in SV fusion, CAZ proteins also coordinate exocytosis-endocytosis coupling (Haucke et al., 2011) by promoting SV protein sorting, transportation from AZs to EZs, and recruitment of endocytosis proteins. Available work focuses primarily on transmitter release—future work is required to explore their roles in SV exocytosis-endocytosis coupling.

EZs have abundant endocytosis proteins such as AP-2, clathrin, dynamin, DAP160, and intersection (Wahl et al., 2013; Gimber et al., 2015). The nanoscale organization of EZs at nerve terminals remains largely unclear. It seems excluded from but adjacent to AZs. An annulus EZ surrounding an AZ is reported at *drosophila* NMJs (Roos and Kelly, 1999); this spatial arrangement between EZs and AZs can facilitate re-capture of SV proteins once they diffuse away from AZs, providing a structural base for the efficient exocytosis-endocytosis coupling (Figure 2D). Based on free diffusion of VAMP2 at a diffusion coefficient (*D*) of 0.2 μm^2^/s at the presynaptic PM (Ramadurai et al., 2009; Gimber et al., 2015; Joensuu et al., 2016), it takes ~2.5 s to diffuse ~1 μm [*t* = *x*^2^/(2 ^*^ *D*)]. In addition to annulus EZs, other forms of EZ organization is also possible in different types of never terminals, including random distribution in the terminals or in patches at peri-AZs (similar to the EZ around PSDs; Lu et al., 2007). EZs appear relatively stable during stimulation and offer a platform where some endocytosis proteins with multiple domains can stabilize other endocytosis proteins and recruit SV proteins. For example, intersectin/DAP160, which has five SH3 domains, binds many proteins including dynamin, synaptojanin, stonin-2, N-WASP, Eps15 homology (EH) domains, and SNAP-25 (Roos and Kelly, 1998; Evergren et al., 2007). Loss of Dap16/Intersectin impairs the FM1-43 loading in fly NMJs and destabilizes dynamin, synaptojanin, and endophilin (Koh et al., 2004; Marie et al., 2004), suggesting its role in stabilizing endocytosis machinery at EZs (Pechstein et al., 2010). However, intersectin-1 KO synapses (Yu et al., 2008) exhibit little endocytosis defect (Sakaba et al., 2013), possibly due to its redundancy in mammals.

Filamentous actin (F-actin) is highly enriched in EZs. An annulus of F-actin is shown to surround the AZ in motor terminals of lamprey (Shupliakov et al., 2002; Bloom et al., 2003; Morgan et al., 2004) and NMJs (Richards et al., 2004), implying its important role in endocytosis. Disruption of F-actin inhibits multiple forms of endocytosis at nerve terminals (Shupliakov et al., 2002; Watanabe et al., 2013b; Wu et al., 2016) (but see Sankaranarayanan et al., 2003). Meanwhile, F-actin also enhances SV replenishment, priming, and fusion at synapses (Sakaba and Neher, 2003; Cingolani and Goda, 2008; Lee et al., 2012, 2013). The dual-role of F-actin in both exocytosis and endocytosis suggests its potential role in coupling these two processes. The underlying details are unclear. F-actin likely enhances SV protein diffusion between AZs to EZs, traps SV proteins in EZs and slows their escaping, and promotes SV scission.

Dynamin is a key component that regulates different endocytosis at nerve terminals. Among three dynamin genes in mammals, Dynamin-1 is the major isoform in neurons (Ferguson et al., 2007). Dynamin-1 KO impairs CME but increases bulk endocytosis ~2-fold (Hayashi et al., 2008; Wu Y. et al., 2014), suggesting its different roles in CME and bulk endocytosis. Dynamin-1 KO calyces alter the short-term plasticity via different mechanisms (Mahapatra et al., 2016; Mahapatra and Lou, 2017). The reduction of synaptic depression selectively at high frequency (>100 Hz) APs (Mahapatra et al., 2016) agrees with the change of endocytosis from CME to the enhanced bulk endocytosis in the absence of dynamin-1 (Mahapatra et al., 2016). Dynamin-1 and-3 double KO exaggerates the phenotypes of single dynamin-1 KO (Raimondi et al., 2011; Fan et al., 2016). In the native brain circuitry, dynamin-mediated endocytosis is required for synapse development and maturation (Fan et al., 2016). Dynamin inhibitors are useful tools in endocytosis studies, but the data interpretation may be more complex than it seems because of its off-target effects (Park et al., 2013; Mahapatra et al., 2016). This sometimes can lead to different conclusions. For example, dynasore blocks ultrafast endocytosis, but it also affects actin (Park et al., 2013), a factor is critical for ultrafast endocytosis (Watanabe et al., 2013b). Moreover, it seems challenging for dynamin molecules to recruit, polymerize and disassemble at fission necks in ultrafast endocytosis, because these processes are known to be slow, ~24 s in CME as measured by direct TIRF imaging in non-excitatory cells (Merrifield et al., 2002; Doyon et al., 2011; Taylor et al., 2011). That is several orders of magnitude slower than 100 ms. The presence of preassembled clathrin coatsd (Wienisch and Klingauf, 2006) can accelerate endocytosis, but clathrin is not required in ultrafast endocytosis at PT (Watanabe et al., 2014) (which agrees with the slow assembly dynamics of clathrin coats; Cocucci et al., 2012). Another possibility is that the dynamics and process of dynamin assembly at fission sites are somewhat different between non-neuronal cells and synapses, such as stronger membrane bending property of dyanmin-1 at synapses than dynamin-2 at non excitatory cells (Liu et al., 2011) and a higher number of dynamin molecules pre-localized at EZs. Therefore, future work is required to address how synapses may use dynamin differently as the fission machinery to regulate different modes of SV exocytosis-endocytosis coupling.

### Local membrane stress

There exists dramatic membrane stress at presynaptic terminals during exocytosis and endocytosis. The local stress can arise from SV fusion itself. SVs are the smallest bi-layer membranous structures that nature can make, with an outer/inner diameter ratio of up to ~4:3 or higher. SV fusion adds more membrane on the inner leaflet than the outer leaflet of the PM, which can produce an asymmetric membrane stress at the local PM. This local stress may delay SV flattening after fusion, promote PM lateral diffusion, and/or enhance PM invagination at EZs (only ~100 nm away from AZs). Furthermore, insertion of SV proteins alters the rigidity of local PM, a factor playing important role in endocytosis (Hassinger et al., 2017).

The local membrane stress likely facilitates the coupling between exocytosis and endocytosis (Figure 2C). The mechanical spread of membrane stress can be fast at AZs and is suitable to trigger those fast forms of endocytosis (e.g., ultrafast endocytosis, K&R, and bulk endocytosis). For example, while it is challenging to utilize traditional molecule signaling as in CME for ultrafast endocytosis, the local membrane stress generated by SV fusion may transfer instantly from AZs to EZs. The downside of this mechanism is that SV components removing from AZs may not be as fast as the force transfer and thus limit the benefit of ultrafast endocytosis during high-frequency neurotransmission.

The characterization of local membrane mechanics is not well-established during SV exocytosis-endocytosis, and it is likely affected by several factors including membrane tension, membrane stiffness, and local force (Hassinger et al., 2017). As a key force generator in cells, F-actin is shown to be required in multiform of endocytosis including ultrafast endocytosis (Watanabe et al., 2013b) and bulk endocytosis (Shupliakov et al., 2002; Holt et al., 2003). It likely generates local vertical force (against the PM) during membrane bending and fission or radial force (parallel the PM plane) at the fission neck (Walani et al., 2015; Hassinger et al., 2017). Endophilin may enhance the local membrane stress (Simunovic et al., 2017) via its curvature-sensing and curvature-generating properties (McMahon and Gallop, 2005). It has been shown to regulate clathrin-independent endocytosis in non-excitatory cells (Boucrot et al., 2015; Renard et al., 2015).

## Perspectives

SV exocytosis-endocytosis coupling has a profound role in synaptic transmission, a process that is essential for neural circuit function and brain performance. Several factors emerge as sensors for endocytosis events, and we are only at an initial stage toward the mechanistic understanding of SV exocytosis-endocytosis coupling at synapses; even how SVs are born under physiological conditions remains a conundrum. The major challenge arises mainly from the complex biophysical features of exocytosis-endocytosis coupling at presynaptic terminals, such as the transient reaction, small structure, and poor accessibility. Despite these hurdles, the field is steadily moving forward with the application of new cutting-edge approaches spanning super-resolution fluorescence microscopy, single SV fusion detection, acute optogenetic manipulations (Ji et al., 2017), flash-freezing EM, and in-situ cryo-EM tomography. With advances in novel techniques and an increasing need to understand synaptic mechanisms, there has never been a better time to engage in investigating this fundamental process of brain function.

## Author contributions

The author confirms being the sole contributor of this work and approved it for publication.

### Conflict of interest statement

The author declares that the research was conducted in the absence of any commercial or financial relationships that could be construed as a potential conflict of interest.
